# 18F-FDG PET/CT as predictive and prognostic factor in esophageal cancer treated with combined modality treatment

**DOI:** 10.1007/s12149-022-01733-9

**Published:** 2022-03-11

**Authors:** Marco Krengli, Eleonora Ferrara, Riccardo Guaschino, Erinda Puta, Lucia Turri, Ilaria Luciani, Gian Mauro Sacchetti, Pierfrancesco Franco, Marco Brambilla

**Affiliations:** 1 Division of Radiation Oncology, University Hospital “Maggiore Della Carità”, corso Mazzini 18, 28100 Novara, Italy; 2 Department of Translational Medicine, University of Piemonte Orientale, via Solaroli 17, 28100 Novara, Italy; 3 Unit of Nuclear Medicine, University Hospital “Maggiore Della Carità”, corso Mazzini 18, 28100 Novara, Italy; 4 Unit of Medical Physics, University Hospital “Maggiore Della Carità”, corso Mazzini 18, 28100 Novara, Italy

**Keywords:** Esophageal cancer, Predictive/prognostic factors, Radio-chemotherapy, Surgery, 18F-FDG PET/CT

## Abstract

**Purpose:**

[18F] fluorodeoxyglucose positron emission tomography/computed tomography ([18F] FDG-PET/CT) is used for diagnosis, staging, response assessment and prognosis prediction in different tumors, but its role in esophageal cancer is still debated. The aim of this study was to evaluate the role of semiquantitative baseline PET parameters as possible prognostic and predictive factors in a series of esophageal carcinomas treated with combined modalities.

**Methods:**

43 patients with esophageal carcinoma were treated with chemoradiotherapy (CRT) followed by surgery in 20 cases and underwent pre-treatment 18F-FDG-PET/CT. Semiquantitative PET parameters were evaluated including Standardized Uptake Value (SUVmax e SUVmean), Metabolic Tumor Volume (MTV) and Total Lesion Glycolysis (TLG) with isocontour of 41 and 50%. Further variables analyzed were gender, primary tumor site, histological type, use of surgery, achievement of a radical resection and the type of chemotherapy regimen. The correlation of all variables with treatment response, loco-regional control (LR), Overall survival (OS) and Disease-Free Survival (DFS) was evaluated.

**Results:**

SUVmax, SUVmean50 and SUVmean41 were significantly higher in node-positive cases and in squamous cell carcinomas. With respect to prognostic factors, MTV was found to be correlated with OS: patients with MTV41 < 11.32 cm^3^ and MTV50 < 8.07 cm^3^ (both *p* values = 0.04) showed better 3-year OS rates (33 vs. 20%). Further factors predicting a better prognosis were the use of surgery and radical resection (R0) (both *p* values < 0.01).

**Conclusions:**

Pre-treatment MTV values were significant prognostic factors for OS, together with the use of surgery and R0 resection in esophageal cancers treated with multimodal therapies.

## Introduction

Esophageal cancer is the seventh most frequently diagnosed cancer and the sixth leading cause of cancer-related deaths in the world, representing a major global health problem [1, 2]. Combined modality therapy has been shown to improve survival in patients with loco-regional disease compared to surgery alone, since a complete (R0) tumor resection cannot be achieved in about 30% (T3)–50% (T4) of cases [3]. Surgery with perioperative treatment is the gold standard treatment for resectable esophageal cancer, and exclusive chemoradiotherapy (CRT) is an alternative option for squamous cell carcinoma [3]. Despite recent improvements in the available treatment modalities, overall survival (OS) remains poor, with a five-year rate of about 15–20% [4]. A better stratification of these patients based on pre-treatment predictive and prognostic factors could optimize treatment strategies. Clinical factors with negative impact on OS include the following: malnutrition, obesity, male gender, cardiological comorbidities, and low socio-economic status [5]. Clinical stage and lymph node involvement are the main tumor-related factors influencing the prognosis of esophageal cancer [6, 7]. Five-year net survival for patients with esophageal cancer shows a difference between Stages 2 and 3, ranging from 30% at Stage 2 to 16% at Stage 3 [8]. Nodal invasion, quantified by absolute number (≥ 4) or ratio of positive nodes ≥ 0.2 compared to all examined lymph nodes, is an independent prognostic factor for OS [5, 7]. Five-year survival rates of 63% for pN0 and 30% for pN + lesions are reported [6, 7]. Moreover, the analysis of markers for treatment response is an important prognostic factor. Positron emission tomography (PET) with 2-deoxy-2-[fluorine-18]-D-glucose (18F-FDG) fused with computed tomography (18FDG-PET/CT) has an emerging role in tumor staging, radiotherapy treatment volume delineation, response assessment and follow-up for several tumors [9, 10]. In esophageal cancer, 18F-FDG PET/CT may lead to a change in treatment management in up to a third of patients, representing the most accurate imaging modality for detecting distant metastases [11–13]. It is still matter of debate if metabolic parameters obtained by PET/CT could have a predictive and prognostic role [14–20]. Some of these parameters, such as maximum standard uptake value (SUVmax), SUVmean, SUVpeaks, tumor functional longitudinal length, metabolic tumor volume (MTV) and total glycolysis (TLG) have been proposed as prognostic indicators in esophageal and other solid tumors [19, 21–23].

The aim of the present study is to evaluate the role of 18F-FDG PET/CT as possible predictive and prognostic factor in a series of patients with esophageal carcinoma, treated with combined modality treatments.

## Materials and methods

Clinical records of patients with histological diagnosis of esophageal carcinoma referred to the Division of Radiation Oncology of the University Hospital “Maggiore della Carità” in Novara, Italy, from 2012 to 2020, were collected. The study was approved by the local review board following the rules of our Institution. All the procedures followed were in accordance with the ethical standards of the responsible committee on human experimentation and with the Helsinki Declaration of 1964 and all subsequent revisions. All patients were informed about the possible use of their documentation in the research study and gave their informed consent prior to their inclusion in the study. Inclusion criteria were as follows: biopsy-proven esophageal carcinoma, age ≥ 18 years, treatment with CRT (neoadjuvant or definitive), and pre-treatment staging including esophagogastroduodenoscopy (EGDS), computed tomography (CT), 18F-FDG PET/CT, ultrasound endoscopy (EUS), and bronchoscopy when indicated. Staging was defined according to the American Joint Committee on Cancer (AJCC) TNM classification 8th ed. [24]. All cases were discussed within a multidisciplinary tumor board comprising pathologists, radiologists, gastroenterologists, surgeons, and radiation and medical oncologists. All patients were treated with combined CRT.

For radiotherapy purposes, each patient had a pre-treatment planning spiral CT (Aquilion Large Bore, Toshiba, Japan). A radiation oncologist with expertise in gastro-intestinal cancers delineated the clinical target volume. Radiation therapy was delivered with a linear accelerator using a 6MV photon beam. Concomitant chemotherapy regimens included weekly carboplatin + paclitaxel, as per the CROSS trial [25] or other platinum-based regimens (cisplatin + 5-fluorouracil, carboplatin monotherapy, FOLFOX). All patients were further assessed at 4–6 weeks after the end of treatment, with EGDS and CT. Those, who underwent neoadjuvant CRT and were deemed unsuitable for surgery after restaging, were candidates for radiation boosts.

Pre-treatment 18F-FDG PET/CT was performed with the same PET/CT scanner (Biograph 16 Hirez, Siemens, Germany) and evaluated both automatically and manually to homogenize all the acquired parameters. PET was performed by OSEM3D reconstruction with 48 equivalent iterations (2i × 24 s), Gaussian smoothing filter 4 mm, FOV 168 × 168, voxel size 4.8 × 4.8 mm, slice thickness 2 mm and emission scan duration from 2 to 3 min per bed position. CT was performed with 100 kV, 36 mAs, slice thickness of 5 mm and FOV 512 × 512. CT images were used for both attenuation correction of PET data and localization of pathological FDG uptake. CT scan was performed without administration of intravenous contrast with a low-dose protocol for CT acquisition. The blood glucose levels of all patients were measured before the injection of 18F-FDG and were < 190 mg/dL. The injected activity of FDG was 3 MBq/Kg and the mean time between injection and acquisition was 81 min (range 57–118 min). For each primary tumor, the following parameters were analyzed: SUVmax, SUVmean, MTV and TLG according to the European Agency of Nuclear Medicine (EANM) guidelines [26]. SUVmax was calculated to determine the 18F-FDG PET activity and recorded using a volumetric region of interest (VOI), positioned around the pathological 18F-FDG uptake in the attenuation-corrected images. Each VOI was checked visually to exclude areas of physiological uptake. MTV was defined as the volume of hypermetabolic tissue with a threshold higher than 41 and 50% of the maximum pixel value in the primary tumor [26]. TLG was defined as the SUVmean multiplied by the MTV. Data were collected to evaluate metabolic parameters detected by 18F-FDG PET/CT prior to CRT in relation with treatment response, assessed with post-treatment imaging (CT and EGDS) and OS, disease-free survival (DFS) and loco-regional control (LRC). In addition to PET parameters, other potential prognostic factors were evaluated as: patient’s gender, primary tumor site, histological type, surgical intervention, margin status (R0 vs. R1/R2), and type of chemotherapy. Post-CRT response assessment on restaging was based on the following criteria: (a) the absence of neoplastic lesions within the radiation field was considered a complete response (CR), (b) a > 30% reduction in the diameters of the neoplastic lesion in the various projections was considered a partial response (PR), and (c) the progression or < 30% diameter reduction for neoplastic lesion was deemed as a non-response (NR). Patients with PR and CR were considered as responders, and those with NR as non-responders. During follow-up, patients underwent the same exams as post-treatment assessment every 6 months for the first 3 years and then every year. Diagnosis of recurrent tumor and distant metastasis was based on clinical and radiological evidence of tumor relapse.

### Statistical analysis

All semiquantitative PET imaging parameters (SUVmax, SUVmean, MTV, and TLG) calculated with isocontours of 41 and 50% were expressed as mean ± standard deviation (SD), median and range. Their normal distribution was assessed by means of the Shapiro–Wilk test. Intra-observer reproducibility was assessed by calculating the intraclass correlation coefficient in the semiquantitative parameters of the PET imaging determined by the same operator in two successive trials on the same dataset.

Two-side Student’s *t* tests for unpaired data (for normally distributed variables) or Mann–Whitney *U* test (for non-normally distributed variables) were used to compare the semiquantitative parameters of the clinical variables categorized as follows: patient’s gender (male vs. female), T-site (3rd upper vs. 3rd middle–lower), lymph node metastases (N + vs. N0), histological type (squamous cell carcinoma vs. adenocarcinoma). Student’s *t* test or Mann–Whitney *U* test were also used as appropriate to compare PET parameters and treatment response, divided into responders (CR + PR) and non-responders (NR).

The correlation of metabolic characteristics with patient’s age and longitudinal extension of the primary tumor was assessed using the Pearson’s correlation coefficient. Only statistically significant correlations were noted in the Tables. To evaluate the association between SUV, MTV, TLG and survival, the median value of every metabolic parameter was used as the cut-off. OS, DFS and LR control rates were calculated using Kaplan–Meier analysis stratified according to cut-off value (median value) and compared using the log-rank test. DFS was defined as the time after treatment during which no sign of any tumor relapse was observed, and LRC was defined as the time after treatment without local or regional tumor relapse. Both were calculated from the end of treatment. Follow-up time was analyzed from the last day of CRT to the date of the last follow-up, recurrence, or death. The same tests were used to compare survival (OS and DFS) among clinical variables categorized as follows: gender (male vs. female), primary tumor site (3rd upper vs. 3rd middle–lower), histological type (squamous cell carcinoma vs. adenocarcinoma), surgery (Yes vs. No and R0 vs. R1/R2) and type of chemotherapy (CROSS trial vs. cisplatin-based). Yates’s chi-squared test was used to evaluate any possible associations between surgery and PET parameters. All statistical analyses were performed using Statistica 6.0 (Stasoft Inc., Tulsa, OK, USA) and statistical significance was set at *p* value ≤ 0.05.

## Results

Forty-three patients meeting the inclusion criteria were enrolled in the current study. Most presented with locally advanced stage (90.7% T3-T4; 83.7% N +). Clinical characteristics of the patients are listed in Table 1. They were treated with external beam RT to a median total dose of 44.0 Gy (range 44.0–59.4 Gy, including boost), with standard daily fractionation (1.8–2 Gy). Seven patients were treated with 3-dimension-conformal RT (3D-CRT), 19 with static intensity modulated radiotherapy (IMRT) and 17 with volumetric modulated arc therapy (VMAT). Radiotherapy was delivered with neoadjuvant intent in 31 patients and definitive in 12. Eighteen patients received concomitant chemotherapy with weekly carboplatin + paclitaxel and 12 received only platinum-based regimens. Treatment modalities are reported in Table 2. According to the assessment criteria, eight patients obtained CR (18.6%), 22 PR (51.2%), and 13 patients NR (30.2%). Twenty of 31 patients treated with neoadjuvant intent were eligible for surgery (6–8 weeks after CRT). Six of the 20 patients (30%) who underwent surgery showed a pathological CR (ypCR), while only two of the 23 non-surgical patients (8.7%) showed a clinical CR complete response (ycCR). The remaining 11/31 patients were not eligible for surgery due to local progression and infiltration of vascular structures in five patients and progression outside the radiotherapy volume in four patients (two at the level of celiac lymph nodes and two with metastases to liver, bone or lung). Intra-observer reproducibility over two trials on the same data showed intraclass correlation coefficients ranging from *R* = 1 (SUVmax) to *R* = 0.98 (SUVmean) demonstrating the excellent reliability of this semiautomatic technique.

**Table 1:** Patients’ clinical characteristics

Characteristics	Value	(%)
Gender (N. of patients)		
Male	33	(76.7)
Female	10	(23.3)
Age (years)		
Median	66	
Range	49–79	
Histotype		
Squamous cell Ca	29	(67.4)
Adeno Ca	14	(32.6)
Esophageal T-site		
Upper third	9	(20.9)
Middle third	17	(39.5)
Lower third	17	(39.5)
Tumor clinical stage (N. of patients)		
cT1-T2	4	(9.3)
cT3-T4	39	(90.7)
Nodal/distant sites clinical stage (No. of patients)		
cN0	7	(16.3)
cN +	36	(83.7)
cM0	40	(93.0)
cM +	3	(7.0)
Post-CRT tumor stage (N. of patients)		
yT0	8	(18.6)
yT1-T2	8	(18.6)
yT3-T4	27	(62.8)
Post-CRT nodal/distant sites stage (N. of patients)		
yN0	13	(30.2)
yN +	30	(69.8)
yM0	35	(81.4)
yM +	8	(18.6)

**Table 2:** Patients’ treatments

Characteristics 1	Value	(%)	Characteristics 2	Value	(%)
RT intent			Combined CT		
Neoadjuvant	31	(72.1)	Pt Based	13	(30.2)
			CROSS	18	(41.9)
Exclusive	12	(27.9)	Pt based	11	(25.6)
			No CT	1	(2.3)
Post-CRT neoad surgery (31 patients)			Surgical radicality		
Not eligible	11	(35.5)	R0	16	(80.0)
Eligible	20	(64.5)	R+	4	(20.0)

The mean values and SD of SUVmax, SUVmean, MTV, and TLG of the baseline [18F] FDG-PET/CT are reported in Table 3, together with the result of the Shapiro–Wilk test for the normality of the distribution.

**Table 3:** 18F-FDG PET parameters of the primary tumor

Variable	Mean (SD)	Median	Minimum	Maximum	Normal distribution	
					W Shapiro–Wilk	*p*
SUVmax	17.6 (7.5)	16.9	4.0	36.1	0.96	0.21
SUVmean 41	10.9 (4.6)	10.7	2.6	21.5	0.97	0.30
MTV 41	18.4 (20.3)	11.3	0.8	12.6	0.65	< 0.001
TLG 41	207.6 (231.4)	105.7	7.8	1263.6	0.74	< 0.001
SUVmean 50	11.9 (4.9)	11.6	2.8	23.3	0.96	0.21
MTV 50	13.2 (14.8)	8.1	0.5	89.8	0.64	< 0.001
TLG 50	164.3 (184.7)	87.3	5.1	992.2	0.75	< 0.001

*SD* standard deviation, *SUVmax* maximum standardized uptake value, *SUVmean* mean standardized uptake value, *MTV* metabolic tumor volume, *TLG* total lesion glycolysis, 41 isocontour 41%, 50 isocontour 50%

The associations between metabolic characteristics and tumor-related variables are summarized in Tables 4, 5. SUVmax (*p* = 0.03), SUVmean50 (*p* = 0.03) and SUVmean41 (*p* = 0.04) values were significantly higher in N + than in N0 cases. SUVmax (*p* = 0.05), SUVmean50 (*p* = 0.04) and SUVmean41 (*p* = 0.03) values were significantly higher in the squamous cell carcinomas than in the adenocarcinomas. MTVs and TLGs values (*p* < 0.01) were found to be directly proportional to the longitudinal extension of the primary tumor, but, in this regard, both were calculated using parameters which derived from the tumor size. Conversely, PET parameters were not influenced by patient age or tumor location in the upper, mid or lower esophagus.

**Table 4:** Metabolic characteristics in relation to patient and tumor-associated variables (Student’s *t* test)

Variable	PET parameter	Mean (SD)		*p* value
		Female	Male	
Gender (Female vs. Male)	SUVmax	20.0 (5.6)	16.7 (7.9)	0.22
	SUVmean 50	13.3 (3.6)	11.3 (5.2)	0.26
	MTV 50	20.9 (26.0)	10.4 (8.4)	0.18
	TLG 50	262.9 (288.9)	127.6 (130.0)	0.12
	SUVmean 41	12.3 (3.4)	10.3 (4.8)	0.24
	MTV 41	28.3 (35.8)	14.7 (11.7)	0.22
	TLG 41	327.4 (363.1)	162.9 (163.5)	0.11
		3rd upper	3rd middle–lower	
T-site (3rd upper vs. 3rd middle–lower)	SUVmax	17.3 (7.4)	18.0 (8.2)	0.82
	SUVmean 50	11.7 (4.9)	12.0 (5.2)	0.87
	MTV 50	12.9 (15.9)	12.4 (10.0)	0.62
	TLG 50	157.2 (189.7)	166.0 (174.6)	0.61
	SUVmean 41	10.7 (4.6)	11.1 (4.8)	0.84
	MTV 41	18.1 (21.9)	16.8 (13.9)	0.83
	TLG 41	198.9 (236.3)	209.5 (225.1)	0.65
		N +	N0	
LND metastasis (N + vs. N0)	SUVmax	18.6 (7.2)	11.8 (6.7)	**0.03**
	SUVmean 50	12.5 (4.7)	8.2 (4.5)	**0.03**
	MTV 50	13.2 (15.5)	10.8 (10.9)	0.64
	TLG 50	166.8 (188.1)	119.3 (173.7)	0.24
	SUVmean 41	11.4 (4.4)	7.6 (4.2)	**0.04**
	MTV 41	18.6 (21.5)	14.1 (13.4)	0.68
	TLG 41	212.5 (237.6)	142.7 (201.9)	0.24
		Squamous cell Ca	Adeno Ca	
Histotype (Squamous cell Ca. vs. Adeno Ca.)	SUVmax	19.0 (7.2)	14.2 (7.3)	**0.05**
	SUVmean 50	12.9 (4.7)	9.6 (4.6)	**0.04**
	MTV 50	14.6 (17.4)	9.1 (5.8)	0.42
	TLG 50	191.2 (210.9)	92.5 (85.2)	0.12
	SUVmean 41	11.8 (4.4)	8.6 (4.3)	**0.03**
	MTV 41	19.7 (23.8)	14.0 (9.4)	0.80
	TLG 41	237.2 (264.9)	126.4 (114.2)	0.18

Statistically significant values are in bold (**p ≤ 0.05**)

**Table 5:** Correlation of metabolic characteristics with patient’s age and longitudinal extension of the primary tumor

Variable	PET parameter	Pearson correlation coefficent	*p* value
Patient’s age	SUVmax	0.11	0.46
	SUVmean 50	0.10	0.54
	MTV 50	− 0.06	0.72
	TLG 50	− 0.01	0.98
	SUVmean 41	0.11	0.48
	MTV 41	− 0.07	0.63
	TLG 41	− 0.02	0.92
Longitudinal extension primary tumor	SUVmax	0.09	0.56
	SUVmean 50	0.09	0.58
	MTV 50	0.65	< **0.001**
	TLG 50	0.63	< **0.001**
	SUVmean 41	0.09	0.57
	MTV 41	0.66	< **0.001**
	TLG 41	0.63	< **0.001**

Statistically significant values are in bold (**p ≤ 0.05**)

**Fig. 1:**
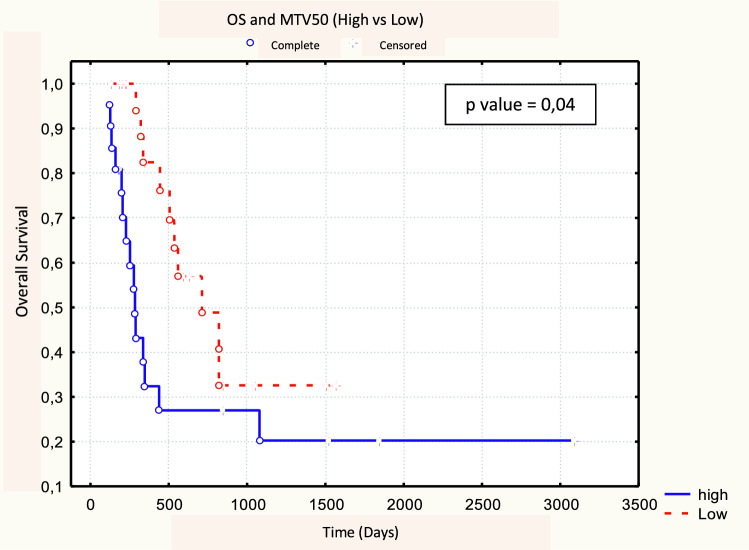
Log-rank test between OS and MTV50 (< 8.1 cm^3^ vs. ≥ 8.1 cm^3^)

**Fig. 2:**
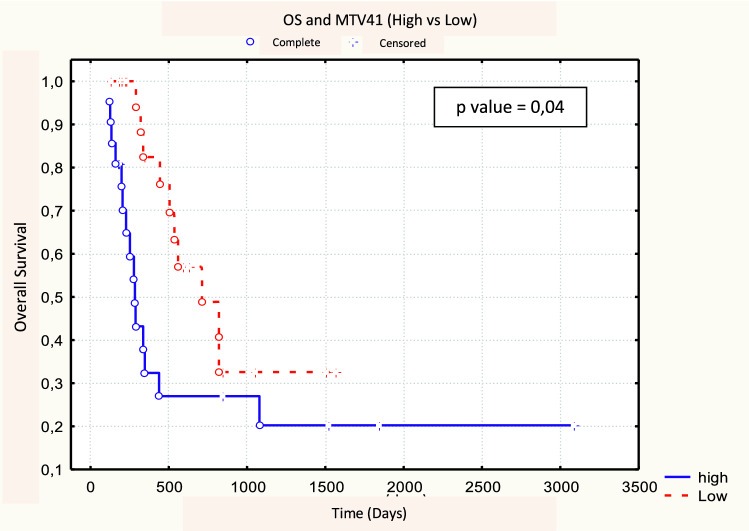
Log-rank test between OS and MTV41 (< 11.3 cm^3^ vs. ≥ 11.3 cm^3^)

**Fig. 3:**
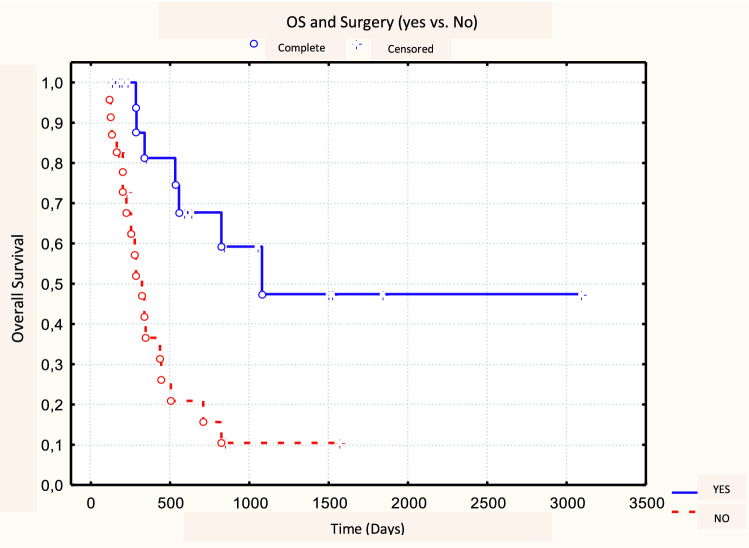
Log-rank test between OS and surgery (Yes vs. No)

**Fig. 4:**
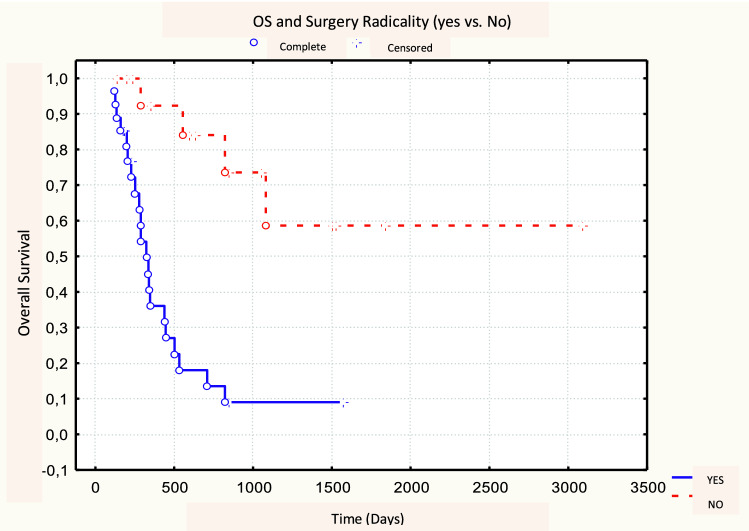
Log-rank test between OS and surgical radicality (R0 vs. ≠ R0)

No significant difference was found between 18F-FDG PET/CT parameters between responders (CR + PR, *N* = 30) and non-responders (NR, *N* = 13). No metabolic parameters with a statistically significant predictive value of response emerged from our analysis (Table 6). After a median follow-up time of 7 months (range 1–98 months), the 3-year OS, DFS, and LRC rates were 32, 35, and 32%, respectively. Of the 30 responding patients (CR + PR) after CRT, 19 (63.3%) developed loco-regional recurrence or distance relapse. At the time of analysis, 32 patients have died (74.4%), 25 of them (78.1%) with progressive disease. Patients with MTV50 < 8.1 cm^3^ (*p* = 0.04) and MTV41 < 11.3 cm^3^ (*p* = 0.04) were correlated to a better OS, in both cases with a 3-year OS rate of 33 vs. 20% (Figs. 1, 2, Table 7). No statistical significance was found amongst the other PET parameters (SUVmax, SUVmean, and TLG) and OS. Among the other potential prognostic factors, surgery (Yes vs. No) and radicality of resection (R0 vs. R1 + R2) were found to be correlated with better OS, (*p* = 0.00074 and *p* = 0.00006, respectively) (Fig. 3 and Fig. 4).

**Table 6:** 18F-FDG PET parameters and response assessment (Student’s *t* test)

PET parameter	Responder’s mean	Non-responder’s mean	*p* value	Responder’s SD	Non-responder’s SD
SUVmax	17.1	18.3	0.63	7.8	7.1
SUVmean 50	11.5	12.3	0.64	5.0	4.7
MTV 50	10.4	18.4	0.10	9.0	22.8
TLG 50	130.7	224.5	0.13	139.3	256.5
SUVmean 41	10.6	11.3	0.61	4.7	4.4
MTV 41	14.6	25.3	0.12	12.4	31.4
TLG 41	166.6	281.0	0.14	173.4	323.9

**Table 7:** 18F-FDG PET parameters in relation to OS, DFS and LR control (log-rank test)

PET parameter	Median	OS	DFS	LR control
		*p* value	*p* value	*p* value
SUVmax	16.9	0.44	0.67	0.87
SUVmean 50	11.6	0.57	0.71	0.90
MTV 50	8.1	0.04	0.09	0.15
TLG 50	87.3	0.08	0.09	0.15
SUVmean 41	10.7	0.57	0.71	0.90
MTV 41	11.3	0.04	0.09	0.15
TLG 41	105.7	0.08	0.09	0.15

To investigate whether surgery influenced the finding of statistical significance of MTV on OS, we analyzed the association between surgery and MTV and found no correlation for these two variables (Yates’s chi-squared test = 2.79, *p* = 0.01).

## Discussion

Despite the improvements in the multimodality treatment of esophageal cancer, the prognosis of this tumor is still poor. Prediction of patient prognosis and survival in the preoperative setting could guide the choice of personalized neoadjuvant/adjuvant treatment strategies. In this regard, metabolic imaging plays a key role in the staging of esophageal cancer and could provide predictive and/or prognostic information. More specifically, SUV max and analysis of tumor response before and after CT/CRT, together with MTV and TLG were suggested as prognostic parameters [19, 20]. Currently, there is no accepted threshold for SUVmax and different definitions of MTV have been used, making it difficult to compare the studies and evaluate the usefulness of MTV [5]. These contrasting results could be related to the inhomogeneity of the 18F-FDG tumoral uptake pattern that is associated not only with increased metabolism, but also with several other parameters such as perfusion, cell proliferation and density, and tissue hypoxia. As a matter of fact, SUVmax reflects only the most active part of the tumor and does not necessarily correlate with the entire tumor burden [27]. Our analysis suggests the MTV to be a prognostic statistically significant parameter. In terms of OS, high values of MTV50 (*p* = 0.04, cut-off 8.0 cm^3^) and MTV41 (*p* = 0.04, cut-off 11.3 cm^3^) correlate with a worse prognosis (3-year survival: 33 vs. 20%). These cut-off levels could be considered the optimal ones emerging from our experience. These results are in line with other data reported in the literature. A study by Chhabra et al. in 51 patients found a correlation between pre-treatment MTV and prognosis in terms of OS, DFS, and LRC; in particular, high values of MTV with cut-off 19 cm^3^ correlated with a worse prognosis [18]. A similar analysis was conducted by Hoseok et al. and Li et al. obtaining a cut-off value for MTV of 1.0 cm^3^ and 10.5 cm^3^, respectively [19, 20].

Literature data indicate TLG also as a prognostic factor: TLG is the product of the MTV and SUVmean and represents both the degree of 18F-FDG uptake and the size of the metabolically active mass [15, 28]. Our data suggest a correlation trend between prognosis in terms of OS and DFS and TLG50 and TLG41 (*p* = 0.08 and *p* = 0.09, respectively). The differences between studies evaluating SUV, MTV, and TLG parameters could be explained by different factors. In most cases, these are retrospective studies with limited case series, hampering accurate comparisons. Moreover, differences in SUV, MTV, and TLG between different tumor histologies are not known in esophageal cancer and may affect the different prognostic roles of MTV and TLG [15]. We did not identify significant predictive parameters; only a trend was found according to the achievement of a complete or partial response (CR + PR) to CRT; responding patients showed lower but non-significant MTV values than those in the non-responder group (mean MTV50 of the two groups: 10.4 cm^3^ vs. 18.4 cm^3^, *p* = 0.10). This fact could be related to an inhomogeneous distribution of the cases with unbalanced patients’ characteristics in the analyzed subgroups (responders vs. non-responders) and to the relatively low number of cases in the non-responder group. On the other hand, only few literature data suggest a predictive role for metabolic parameters based only on pre-treatment PET/CT, without a comparison of post-CRT PET/CT. Levine et al. in a series of 64 patients with esophageal carcinoma reported a possible role of SUVmax as a predictor; in this case, patients with pre-treatment SUVmax > 15 responded better to treatment [14]. The identification of predictive parameters of response to therapy could come from studies comparing systematically the metabolic parameters both before and after CRT [29]. Another well-known prognostic factor is surgery: in our series, patients eligible for surgery showed better survival in terms of both OS (survival 3-year 48 vs. 10%, *p* = 0.00074), and DFS (3-year survival 56 vs. 10%, *p* = 0.0014). The same applies to those patients who showed a pathological complete response, in terms of both OS (3-year survival of 59 vs. 10%, *p* = 0.00006) and DFS (3-year survival of 67 vs. 18%, *p* = 0.0001). Of note, surgery did not influence the value of MTV as a prognosticator for OS as shown in our analysis by Yates's chi-squared test. The main limitations of our study are related to its retrospective nature, the relatively limited number of patients and the lack of data regarding post CRT PET/CT response.

## Conclusions

The data here presented suggest a prognostic value for preoperative MTV, independent of the use of surgery, and support the hypothesis that patients with lower preoperative MTV values could have a better prognosis even after radical CRT regimens. These findings need to be validated within prospective studies on a larger cohort of patients, to investigate more in-depth prognostic factors to improve treatment strategies in esophageal cancer. Further investigations by omics studies could open the way for more customized treatment, considering tumor signatures related to response to specific treatment approaches.

## References

[1] Bray F, Ferlay J, Soerjomataram I (2018). Global cancer statistics 2018: GLOBOCAN estimates of incidence and mortality worldwide for 36 cancers in 185 countries. CA Cancer J Clin.

[2] Rustgi AK, El-Serag HB (2014). Esophageal carcinoma. N Engl J Med.

[3] Lordick F, Mariette C, Haustermans K (2016). Oesophageal cancer: ESMO clinical practice guidelines for diagnosis, treatment and follow-up. Ann Oncol.

[4] Polednak A (2003). Trends in survival for both histologic types of esophageal cancer in US surveillance, epidemiology and end results areas. Int J Cancer.

[5] Vendrely V, Launay V, Najah H (2018). Prognostic factors in esophageal cancer treated with curative intent. Dig Liver Dis.

[6] Bouvier AM, Binquet C, Gagnaire A (2006). Management and prognosis of esophageal cancers: has progress been made?. Eur J Cancer.

[7] Mariette C, Piessen G, Briez N, Triboulet JP (2008). The number of metastatic lymph nodes and the ratio between metastatic and examined lymph nodes are independent prognostic factors in esophageal cancer regardless of neoadjuvant chemoradiation or lymphadenectomy extent. Ann Surg.

[8] Cancer research UK (2015). [Online] Available at: https://www.cancerresearchuk.org/health-professional/cancer-statistics/statistics-by-cancer-type/oesophageal-cancer/survival. Accessed 15 Dec 2021.

[9] Krengli M, Milia M, Turri L (2010). FDG-PET/CT imaging for staging and target volume delineation in conformal radiotherapy of anal carcinoma. Radiat Oncol.

[10] Deantonio L, Krengli M, Turri L (2018). Does baseline [18F] FDG-PET/CT correlate with tumor staging, response after neoadjuvant chemoradiotherapy, and prognosis in patients with rectal cancer?. Radiat Oncol.

[11] Barber TW, Duong CP, Leong T (2012). 18F-FDG PET/CT has a high impact on patient management and provides powerful prognostic stratification in the primary staging of esophageal cancer: a prospective study with mature survival data. J Nucl Med.

[12] Goel R, Subramaniam RM, Wachsmann JW (2017). PET/Computed tomography scanning and precision medicine: esophageal cancer. PET Clin.

[13] Meyers B, Downey R, Decker P (2007). The utility of positron emission tomography in staging of potentially operable carcinoma of the thoracic esophagus: results of the American college of surgeons oncology group Z0060 trial. J Thorac Cardiovasc Surg.

[14] Levine E, Farmer M, Clark P (2006). Predictive value of 18-fluoro-deoxy-glucose-positron emission tomography (18F-FDG-PET) in the identification of responders to chemoradiation therapy for the treatment of locally advanced esophageal cancer. Ann Surg.

[15] Hong J, Kim H, Han E (2016). Total Lesion Glycolysis Using 18F-FDG PET/CT as a prognostic factor for locally advanced esophageal cancer. J Korean Med Sci.

[16] Omloo J, van Heijl M, Hoekstra O (2011). FDG-PET parameters as prognostic factor in esophageal cancer patients: a review. Ann Surg Oncol.

[17] Schroer-Gunther M, Scheibler F, Wolff R (2015). The role of PET and PET-CT scanning in assessing response to neoadjuvant therapy in esophageal carcinoma. Dtsch Arztebl Int.

[18] Chhabra A, Ong LT, Kuk D (2015). Prognostic significance of PET assessment of metabolic response to therapy in oesophageal squamous cell carcinoma. Br J Cancer.

[19] Hoseok L, Kim K, Kim SJ (2012). Prognostic value of metabolic volume measured by F-18 FDG PET-CT in patients with esophageal cancer. Thoracic Cancer.

[20] Li Y, Zschaeck S, Lin Q (2019). Metabolic parameters of sequential 18F-FDG PET/CT predict overall survival of esophageal cancer patients treated with (chemo-) radiation. Radiat Oncol.

[21] Hyun SH, Choi JY, Shim YM (2010). Prognostic value of metabolic tumor volume measured by 18F-Fluorodeoxyglucose positron emission tomography in patients with esophageal carcinoma. Ann Surg Oncol.

[22] Lee HY, Hyun SH, Lee KS (2010). Volume-based parameter of 18 F-FDG PET/CT in malignant pleural mesothelioma: prediction of therapeutic response and prognostic implications. Ann Surg Oncol.

[23] Dibble EH, Alvarez AC, Truong MT (2012). 18F-FDG metabolic tumor volume and total glycolytic activity of oral cavity and oropharyngeal squamous cell cancer: adding value to clinical staging. J Nucl Med.

[24] Amin B, Edge S, Green F (2017). AJCC cancer staging manual.

[25] Eyck BM, van Lanschot JJB, Hulshof M (2021). Ten-year outcoe of neoadjuvant chemoradiotherapy plus surgery for esophageal cancer: the randomized controlled CROSS tiral. J Clin Oncol.

[26] Boellaard R, Delgado-Bolton R, Oyen W (2015). FDG PET/CT: EANM procedure guidelines for tumour imaging: version 2.0. Eur J Nucl Med Mol Imaging.

[27] Vesselle H, Schmidt RA, Pugsley JM (2000). Lung cancer proliferation correlates with (F-18) florodeoxyglucose uptake by positron emission tomography. Clin Cancer Res.

[28] Sonoda A, Yoshida N, Shiraishi S (2021). Total lesion glycolysis ratio in positron emission tomography/ computed tomography images during neoadjuvant chemotherapy can predict pathological tumor regression grade and prognosis in patients with locally advanced squamous cell carcinoma of the esophagus. Ann Surg Oncol.

[29] Borggreve AS, Goense L, van Rossum PSN (2020). Preoperative prediction of pathologic response to neoadjuvant chemoradiotherapy in patients with esophageal cancer using 18F-FDG PET/CT and DW-MRI: a prospective multicenter study. Int J Radiat Oncol Biol Phys.

